# Global Crises and the Role of BISE

**DOI:** 10.1007/s12599-020-00657-w

**Published:** 2020-06-23

**Authors:** Oliver Thomas, Simon Hagen, Ulrich Frank, Jan Recker, Lauri Wessel, Friedemann Kammler, Novica Zarvic, Ingo Timm

**Affiliations:** 1 Osnabrück University, Osnabrück, Germany; 2 University of Duisburg-Essen, Essen, Germany; 3 University of Cologne, Köln, Germany; 4 University of Bremen, Bremen, Germany; 5 German Research Center for Artificial Intelligence (DFKI), Osnabrück, Germany; 6 Trier University, Trier, Germany; 7 German Research Center for Artificial Intelligence (DFKI), Trier, Germany

## Introduction

*Oliver Thomas, Osnabrück University and German Research Center for Artificial Intelligence (DFKI). Simon Hagen, Osnabrück University*

The COVID-19 pandemic has surprised the modern world and has presented challenges on an unprecedented scale. Within a few months of the first case being reported at the end of 2019, almost every country in the world is now affected (WHO 2020). Restrictions on public life were not made fast enough and, in some cases, were not sufficient to stop the global spread. Here we see the dark sides of globalization. The crisis is coming with an intensity undreamt of for today’s generations, affecting all areas of life and, at least temporarily, fundamentally changing them. The social, economic and political effects are clearly noticeable and solutions are being sought everywhere to mitigate the crisis and both its direct and indirect consequences.

Upon observation, digitization and digitalization can be seen as an essential part of the solution strategies being discussed to handle the crisis and that associated technologies and concepts are in great demand. For example, the great need for remote communication and collaboration is creating a boom for relevant providers such as Microsoft Teams or Zoom, which have seen an increase from 10 to over 200 million active participants (Yuan 2020) through the provision of rudimentary communication solutions. Even emerging technologies, like blockchain, are being used as a solution strategy through initiatives such as the MiPasa^1^ platform, which is being developed by the World Health Organization (WHO) in cooperation with large technology companies. However, for the implementation of these approaches, not only the technical perspective, but also their integration into economic and social contexts is of great importance (Leidner et al. 2009).

The perception of this challenge in terms of socio-technical systems consisting of people, tasks and technologies and their interrelationships is obvious at this point. It allows conclusions to be drawn about the role that Business and Information Systems Engineering (BISE) research can play in situations such as the current one. Or perhaps must play? As an established research discipline that combines business administration, economics and computer science, BISE can build upon a broad canon of methods and theories (e.g., Bichler et al. 2016), which today has an even larger number of interfaces with other disciplines. This interdisciplinary and application-oriented approach, however, raises the question of how this multitude of perspectives can be combined in a single academic discipline to form a common understanding – or if this is even necessary. Apparently, BISE cannot master the challenges on its own. Yet emerging as a cohesive community can be beneficial in different ways to provide direct or indirect support, specifically in the context of the current crisis, to overcome the manifold challenges.

The distinction between direct and indirect support seems suitable, since BISE can not only contribute to mitigate the consequences of the crisis, but also directly address its challenges. The ‘investigation and control of risks in global networks’ identified by Mertens and Barbian (2015) as the most mentioned grand challenge for the BISE discipline showcases this direct involvement very aptly. It can, on the one hand, be expressed exemplarily in the investigation or design of information systems for crisis management (Pan et al. 2012). On the other hand, the BISE discipline can contribute to the indirect management of crisis situations through research that has so far been strongly application-oriented and design-driven, e.g., in a business context. In relation to this, the intra- and inter-company process and information systems must be adapted to the new challenges in order to remain capable of acting to a reasonable extent despite new legal and social requirements.

The BISE discipline thus can play a two-part role in crisis situations which it can live up to better with a common perception of its unifying core ideas. This has already become apparent when looking at ‘classic’ BISE topics such as information management, business modeling and process management and is currently being discussed and developed for topics such as value creation systems, collaboration and cooperation systems, health applications and the use of artificial intelligence to name a few. Especially in crisis situations, where prompt action is required, this can mediate the in-time application of proven concepts but holds the risk of one-dimensional and short-ranging resolutions.

In order to discuss these and subsequent challenges we invited researchers from different fields of BISE research to critically examine the role of our discipline from their perspective and to discuss arising opportunities and potentials. In this way we cope with the interface-oriented focus of our discipline and identify barriers and approaches to reasonable solutions. Of course, these aspects do not relate exclusively to the current situation, but are also transferable to other exceptional situations and thus pave the way for a more consistent understanding of our discipline in such situations.

Ulrich Frank from the University of Duisburg-Essen takes the perspective of conceptual and enterprise modeling in his essay. He derives proposals for disaster preparedness and describes how methods and concepts of modeling can be applied.

Jan Recker from the University of Cologne addresses the questions of how crises can be better understood and their consequences mitigated. In doing so, he specifically considers the design- and empirically-oriented aspects of the BISE discipline.

Lauri Wessel from the University of Bremen discusses the perspective of digital health under the influence of the current crisis situation. In particular, he focuses on the role of digital transformation on an organizational and societal level.

Friedemann Kammler and Novica Zarvic from the Smart Enterprise Engineering research department of the German Research Center for Artificial Intelligence (DFKI) shed light on the interplay of quick responses and substantiated actions in crisis situations. They propose possible response strategies with a special focus on value networks.

Ingo Timm from the University of Trier reports on the potentials of artificial intelligence in crisis response and the interrelationship with BISE.

## How can Business Informatics Contribute to Disaster Preparedness?

*Ulrich Frank, University of Duisburg-Essen*

The older among us will remember at least one negative consequence of the 9–11 disaster. An outrageous event, which no one would ever have thought possible, not only promoted a general concern for public safety, but also led to many companies suddenly seeing the loss of resources through terrorist attacks as a real threat. Risk management, which until then had led a rather shadowy existence in most organizations, made it to the top of the list of managerial attention. “Business continuity management” quickly became a buzzword, and it did not take long until textbooks were published that promised to guide organizations by developing and implementing business continuity plans (e.g., Dougthy 2001; Elliott et al. 2002). Also, numerous consultancy firms expanded their service portfolio to include risk analysis methods and the development of business continuity plans. Protecting IT infrastructures was regarded as especially important, because it was obvious that the failure of mission critical IT systems was likely to have devastating effects on business operations (e.g., Wieczorek 2002; Geuhs 2003).

The business information systems research agenda did not remain unaffected either. Various approaches aimed at supplementing business process models or enterprise models with concepts to represent risks and possible measures to enable the continuity of operations in case of an emergency (e.g., Neiger et al. 2006; Strecker et al. 2011). Later, when the images of collapsing skyscrapers slowly faded out of collective memory, and under the impression of a growing number of spectacular IT security breaches, attention moved to the protection of IT assets. The ISO/IEC standard series 27,000 is a reflection of those concerns. It comprises guidelines for coping with various kinds of IT and information related risks. The threats the standard focuses on include natural disasters, physical damage, technical failures, exposure of information, and illegitimate action such as terrorist attacks, hacker attacks, espionage, etc. (Klipper, p. 47). Global pandemics are not on the list. Therefore, an ISO-certified business continuity plan would not be of noteworthy help in the face of the current crisis.

Against this background, the question is if and how business informatics could contribute to prepare organizations for coping with disasters. It is beyond my competence to give a convincing answer to this question; and it would be presumptuous for our discipline to promise a comprehensive solution. I can only offer a few thoughts on possible options, which are primarily related to the protection of information systems and their potential to support coping with disasters. My focus is on conceptual modelling. Conceptual models are an indispensable instrument to analyze and assess vulnerabilities and threats. The abstractions models are based on help us move irrelevant aspects out of the picture to see more, that is, to focus on the foundational aspects of an organization, the necessary resources and operations, which must be preserved for an organization to survive. In addition, models are required to develop and communicate *possible* future scenarios. This has been known for some time. Various methods for risk analysis and damage control are based on conceptual models or on enterprise models in particular (Neiger et al. 2006; Strecker et al. 2011; Goldstein and Frank 2016). Since none of these methods accounts for the specific threats generated by a global pandemic, it might appear obvious to extend them with further concepts. However, before we start working on modelling methods that support the analysis of risks caused by pandemic diseases, we should take our time to reflect upon the lessons from the past.

In the face of the current crisis, it is seems advisable for academia to show humility and to be reluctant to give rash advice. Nevertheless, I dare to share a few -hopefully not too premature- attempts to develop a rational and constructive perspective on disaster preparedness.

*Avoid the obvious concentration on the specific peculiarities of the latest disaster*. After 9/11, measures to protect resources against physical destruction were at the center of many business continuity plans. Physical protection of data centers, however, did not provide protection against the shock waves created by the financial crisis in 2008, nor are they of substantial use in the current crisis. Note that this proposal does not intend to mitigate previous threats. They should stay on the list. It only warns of the known framing effect a recent state of emergency may produce.

*Systematic abstraction supports coping with contingencies*. Since we cannot expect to know the shape of the next crisis, it is useful but not sufficient to account for multiple possible scenarios. In order to prepare for the unknown, systematic abstraction is a powerful tool. The more actual resources, products, capabilities and operations are abstracted away, the wider the space opens for alternative instantiations in terms of re-framing: “Reframing operates on the level of meta reality, where […] change can take place even if the objective circumstances of a situation are quite beyond human control.” (Watzlawick et al., p. 97) If, for example, materials, products and production lines are modelled on a more abstract (meta) level, it would ideally be possible to quickly create new instances that would allow the mass production of products required to cope with a state of crisis.

*Open your mind for even the unimaginable, but avoid a race for the most apocalyptic scenario*. Preparedness requires imagination. Therefore, it seems reasonable to develop scenarios of possible future threats on a regular basis. The design of such scenarios should be guided by a dedicated modelling method to emphasize focus and efficiency, but also benefit from the freedom of unrestricted imagination. Nevertheless, accounting for primarily apocalyptic scenarios should be avoided, since seemingly minor events may have devastating effects on particular businesses too.

*There is a need for separation of concerns and for bundling resources*. The development of conceptual models to enable the systematic analysis of threats and the evaluation of relevant probabilities may require an effort that goes beyond the capabilities of many organizations. Furthermore, the evaluation of certain hazards as well as the development of possible counter-measures may not be part of an organization’s responsibility. Therefore, it would be reasonable to develop different layers of models, which, ideally, would be integrated. At the top level, national or cross-national bodies would model scenarios that describe how certain parts of society might be affected and what counter-measures seem reasonable. On lower levels, reference models for entire industries would allow for the reduction of modelling costs and the improvement of model quality at the same time. Models of particular organizations would then only be required as a supplement to account for specific peculiarities.

*Model engineering is not enough*. The analysis and design of conceptual models demands a systematic, rational approach. However, an engineering approach as well as conceptual models in general are not sufficient. Preparedness also demands fostering a culture of resilience that emphasizes corporate spirit, empathy, and responsibility.

## Aiding Citizens in Crisis Situations through State-Tracking and Sensemaking: Lessons Learnt from BISE Research on Representations and Sustainability Transformations

*Jan Recker, University of Cologne*

It is probably fair to say that many of us have been surprised – if not shocked – by the onset and dynamics of the current crisis surrounding the COVID-19 pandemic. I was, at least. I now organize my life around remote work from home, physical distancing and the parenting of kindergarten-age kids. All the while, I follow the ongoing trajectory of the pandemic from the viewpoint of an empirical scientist (What data do we have? What does it measure? What conclusions can we draw?) and as a BISE researcher (What is the role of digital technologies in handling this crisis? How do information systems help society, government, companies and citizens at large?).

The question of how information systems can help in crisis situations is worth asking. It allows BISE research to step out of the corset of business schools in which many of our departments reside. It allows the bringing together of empirically-oriented and design-oriented BISE research. We simply need to both understand the crisis and develop ways to handle it. Traditional separations between methodologies (such as design and behavioral research), disciplinary boundaries (e.g., between health, economics, and information systems) and outcomes (explanation versus construction) appear both irrelevant and blurred at the same time. The world is looking for help and it looks *in particular* to digital technologies to help:

- Understand the crisis (e.g., through information systems that trace and collect data about the pandemic) and
- Mitigate its consequences (e.g., through information systems that facilitate remote collaboration and virtual education).

In my opinion, we possess an excellent theoretical and methodical repertoire in BISE research to tackle both challenges. Leveraging this repertoire is our responsibility and opportunity.

**How IS can help better understand crises.** There is a long-standing research program about the role of information systems in representing the world around us (Burton-Jones et al. 2017; Recker et al. 2019). Its fundamental idea is that information systems that faithfully (i.e., completely and clearly) represent real-world phenomena will be useful because they provide a more cost-effective way of observation than tracking the focal real-world phenomena directly (Weber 2003).

The current public debate about platforms such as the RKI COVID-19-Dashboard (https://corona.rki.de) or the COVID-19 Global Map (https://coronavirus.jhu.edu/map.html) are a case in point. Both platforms are IS designed to faithfully represent the existence and spread of the pandemic. In political discourse and public media, we are currently witnessing how their usefulness is widely debated. This debate about their usefulness is really about their representational faithfulness, and the lamented issues sociotechnical at their roots. How accurate are the data? How timely? What are the time lags in the line of reporting (e.g., every weekend we see dropping numbers of new infections, mainly because of closed healthcare institutions)? What do we not measure accurately (e.g., number of negative tests) and to what extent is technical infrastructure to blame?

To me, these real-world cases show the same fundamental disproportion in terms of what is important in representation in the academic discourse in BISE over the past thirty years: the challenge is not about *representing things and their states* (e.g., countries and their mortality rates). Instead, it is about faithfully tracking *changes of states and events* that cause these. Wand and Weber (1995) developed such a model to faithfully track events and changes over time, their so-called state-tracking model. It stipulates four criteria (Recker et al. 2019, pp. 769–770) that an IS representation of a phenomenon (e.g., a pandemic or other crisis) must meet to ensure that the representation provided by the IS stays accurate and complete – even as things in the real-world change. Some real-world changes that could be seen would be citizens that become infected, are hospitalized, or develop immunity. It must also reflect external events hat change the state of things, some examples being public measures such as home lockdowns, the availability of medication or vaccination, or the expansion of hospital beds in ICUs. We have conditions for such a system (e.g., for mapping, sequences, external events), but as we noted earlier (Recker et al. 2019, p. 753), the state tracking model’s “uptake has been too limited to evaluate its premises”. We simply have not yet built or evaluated state tracking systems systematically enough. Thus, the opportunity is now to help institutions to develop useful representational systems that can faithfully *model and track* all states and events relevant to understanding the pandemic and its future trajectory. Because we have dealt with representations since the beginning of computing, we should be well placed to explain and develop more faithful (and hence more effective) information systems. Systematic evaluation of the effective use of such system could not only help policy makers and crisis managers but also inform the future theoretical development of our own theories of representation, by either refuting, accepting or modifying the theorized criteria for faithful state-tracking. Both success and failure would advance the BISE research program (Burton-Jones et al. 2017).

**How IS can help implementing mitigation strategies.** A second parallel I see is to the BISE research stream that examines IS solutions for environmental sustainability (Gholami et al. 2016). This research tradition has already shown that IS can help make wicked grand challenges such as climate change tractable (Ketter et al. 2016) and effectuate behavioral change in consumers, workers, and other societal groups (Kahlen et al. 2018; Tiefenbeck et al. 2018).

One core insight in this research has been that IS help individuals and collectives with *sensemaking*, i.e. framing, interpreting, and understanding multi-layered and complex issues to create a launchpad for transformation of behavior (Weick et al. 2005). BISE research has both studied IS-enabled sensemaking (Hasan et al. 2017; Seidel et al. 2013) and created new information systems to support sensemaking (Degirmenci and Recker 2018; Seidel et al. 2018).

As the public debate about “restarting” business and society in the wake of the pandemic hones in on using mobile applications for tracking and tracing, I believe BISE research can expand the focus and utility of such technologies, to not only cover representation (tracking) and state-tracking (tracing) but also sensemaking; for example, through features that allow for *reflective disclosure* (to allow citizens to gauge and adapt their behavior by obtaining direct feedback and comparing it to others) and *information democratization* (to allow citizens to engage in public debate and inform strategic choices about lockdown measures and exit strategies). The feasibility and efficacy of such IS solutions has been demonstrated in the context of sustainability transformations. Therefore, I believe similar principles can help the design of digital technologies (mobile or otherwise) that guide citizens to engage in the type of responsible and solidary behavior the world rightfully expects from all of us.

These two examples are meant to demonstrate the immense opportunity for BISE research to live up to the demands it has repeatedly placed upon itself: to be both rigorous and relevant, to offer both explanations and solutions to societal challenges, and to become a reference discipline in its own right. We have not had a situation that more fundamentally and boldly presents this challenge to our field. It is here now, and the onus will be on us to deliver. And I do hope this means stopping the chase after yet another publication in yet another journal and instead focusing our expertise and experience on bringing both new IS solutions, as well as new knowledge about fundamentally sociotechnical problems of crises to those that need them – policy makers, public departments and all fellow citizens. These audiences do not want an IS paper on Corona, they want IS knowledge to solve this crisis and prevent the next.

## What COVID-19 may Mean for Digital Health

*Lauri Wessel, University of Bremen*

Looking at the number of lectures that I have to organize as live sessions this week, it is easy to see that the corona virus has digitally transformed my life in no time. As a researcher working on digital health, I am wondering whether the digital transformation of health care will also be accelerated by the unfolding pandemic.

My overall suggestion in this discussion section is that the COVID-19 pandemic has inflicted massive pressure to digitally transform processes on policy, organizational, and individual levels. We as scholars have an opportunity to partake in designing and managing these transformations if we take seriously the interplays between the technical structure of (business) processes and the social contexts in which they operate (Baiyere et al. 2020; Beverungen 2014). Thus, this discussion also serves as a general plea for conceptual innovation at the intersection of BISE/IS and organization theory literatures (Holeman and Barrett 2018; Orlikowski and Barley 2001; Rothe et al. 2020; Sein et al. 2011). In what follows, I will briefly discuss digital transformation at the various levels I just mentioned and I will sketch out some thoughts about how the “engaged scholarship” (Mathiassen and Nielsen 2008; van de Ven 2007) inherent to our field can help to respond to COVID-19.

It is easy to sense that COVID-19 has set into motion substantive digital transformation on the policy level. Several countries, as well as major companies, are reportedly working on apps to manage the spread of the pandemic. These efforts are accompanied by other non-pharmaceutical interventions (NPIs) such as the closing of schools. While appearing to be different at first glance, these types of interventions are interrelated because data on the spread of the pandemic has become central to political decision-making. The BISE/IS field can play a very important role in mindfully implementing initiatives such as these. There is already work in our field that shows that potential. Mirbabaie et al. (2020) recently put forth that authorities and policy makers could respond to COVID-19 more effectively by providing early information about arising problems to social networks. Likewise, Feuerriegel and colleagues recently estimated the impacts of non-pharmaceutical interventions (NPIs) on reported COVID-19 cases (Banholzer et al. 2020). These authors indicated that the impacts of closing schools and kindergartens were comparably modest. Yet, many in and beyond our field have been affected by the closing of these institutions quite tremendously. The more general issue arising from this observation is that data science, statistics, analytics, artificial intelligence and the capabilities to master them are clearly needed by policy makers and our field can provide them. Moreover, given the centrality of data, questions about the design of appropriate NPIs emerge. In my view, one challenge that lies at the forefront of designing digital interventions for COVID-19 is to bring into balance the potentially diverging aims of far-reaching data aggregation and analysis versus securing data privacy. While I cannot provide a finite answer on how to do this, it is my belief that the BISE/ IS field is in a very good position to respond to this challenge that arises because technical potency and social contexts partially diverge. Taking into account organizational theory on legitimacy of digital solutions could be of help (Constantinides and Barrett 2015; Hinings et al. 2018; Suchman 1995; Wessel et al. 2017) when used as ‘kernel theory’ in design science research.

The digital transformation of organizations is an obvious concern for our field; as recent conference themes and the increasing amount of publications on the matter suggest (Vial 2019; Wessel et al. 2020; Yoo et al. 2010). Organizations of different kinds populate the health care industry and I will focus on hospitals here. Horrifying pictures have been broadcast globally in the context of COVID-19. Clinicians have been forced to care for enormous amounts of patients and have been forced to make tough ethical decisions. The sheer amount of hospitalizations calls for hospitals to be mindful about managing processes in such a fashion that they can handle a maximum number of patients. Yet demand for adequate business process management as such is not new. However, it is the demand to arrive at suitable processes extremely quickly that characterizes this pandemic. It is here where I see strong potentials for BISE/IS to contribute. We know from years of research on implementation of enterprise resource planning systems in hospitals that this is a challenging task (see, for example, Davidson 2002; Kohli and Kettinger 2004). Health care is a heterogeneous domain where different expert professions interact during the treatment of patients (Barrett et al. 2012; Oborn et al. 2011). This is important for responding to COVID-19 since patients with multiple conditions are most at risk and particularly these patients need to be treated by multiple professionals. Efforts to manage and design interventions that enable hospitals to redesign their processes adequately will profit from literature on pluralistic organizations (Berente et al. 2019; Berente and Yoo 2012; Seidel and Berente 2013) because it can explain why health care professionals respond to IT implementation in different ways (Faik et al. 2020; Hansen and Baroody 2020). It could well be that COVID-19 eases the problems associated with implementing health care IT in hospitals as the pandemic seems to create a shared awareness of the importance of IT among different health professionals. On the other hand, COVID-19 could also reinforce these problems as they oftentimes result from professional autonomy and expert knowledge (Goodrick and Reay 2011). What will happen and under what conditions is but one important question for BISE/IS. Also, from a design perspective, interesting questions could relate to how to develop scientific methods for designing IS that respond to the demands of heterogeneous professionals at a very quick pace. Research projects often take two to three years and this is arguably too long for suitable responses to COVID-19 to be implemented.

Finally, COVID-19 is potentially linked to the digital transformation of health-related behaviors on the individual level; an area that IS research has begun to explore just recently (Baskerville 2011; Dadgar and Joshi 2018; Wessel et al. 2019). Mobile apps are at the forefront of interventions into the pandemic. Not only do they enable the tracing of individuals but they also enable self-testing or the receiving of test results. This coincides with a development where apps are increasingly used for the self-management of one’s general health. One important topic that arises would be to assess the acceptance of apps that are supposed to keep the pandemic at bay given potential concerns regarding data privacy. Furthermore, a general question that arises from COVID-19 is whether it may have effects on the acceptance of prevention. Prevention, as opposed to curation or self-management, is supposed to avoid or delay conditions, which is different from mitigating their impacts. Prevention has been a top priority for policy makers for years but individuals often struggle to understand why prevention matters. For example, in an ongoing design science study, we evaluated a smart service to promote prevention. Respondents were around 20 years old and frequently told us “this is not valuable, I am not sick”. Yet many of the apps that are currently developed to respond to COVID-19 are about *preventing* its spread. Will this increase the likelihood that individuals enact prevention? How should the according tools be designed? What are their long-term impacts? I am looking forward to studies that address these and related questions.

## Responsiveness or Substantiated Action? Drawing on the Facets of BISE in Global Crises

*Friedemann Kammler, Novica Zarvic, German Research Center for Artificial Intelligence (DFKI)*

### BISE Meets Global Crises

The COVID-19 pandemic shows the effects that global crises impose on society across nations and how they spark interest in research that serves as a source for well-founded explanations and solutions. Separate from the current focus, global crises are by far not limited to health issues, as many other examples quickly come to mind. For instance, one could name the climate crisis, the financial crisis in 2008, armed conflicts that led to refugee crises or even the recently emerged resource crisis caused by an overproduction of oil. At first glance, each of the aforementioned crises appears to be mainly linked to a single appropriate discipline. Yet, it is obvious that accompanying problems demand responses that need to be crafted on a wider scale. While a discipline is a cognitive and social entity that mostly arose historically and is clearly distinguishable from other scientific areas (Defila and Di Giulio 1998), strict demarcations fade when solutions apply knowledge from different fields – in short, when global crises are approached in an interdisciplinary fashion.

The Corona crisis, hence, is not a secluded medical challenge, as it also raises extensive economic, social and psychological issues. From our point of view, the resulting complexity of finding adequate answers reopens the question whether responsiveness has to be prioritized in order to remedy the situation, or whether answers must be substantiated in order to apply scientific thoroughness (van der Walle and Turoff 2007, p. 30).

We asked ourselves how Business Information Systems Engineering (BISE) can contribute to crisis response against the background of these preliminary considerations. Although BISE also makes various considerable contributions in other fields, we will narrow down our perspective and limit it to problems and solutions that impact businesses. Other than that, our goal is rather pragmatic – to discuss current examples and learn actionable guidelines along three central avenues.

### First Aid

Remaining operational is key in global crises and requires businesses to react immediately to turning tides. At the onset of the pandemic, companies faced the disruption of processes and value networks. This was caused by both the crisis itself and the countermeasures that needed to be taken. “Social distancing” was one example that was implemented nation-wide as an emergency measure to reduce personal contact and to simplify infection chains in order to interrupt them more effectively. The broadly discussed flip side of the coin was a tremendous impact on business activities. These ranged from the introduction of remote work from home to the global discontinuation of services such as after sales. Many companies reacted to this with ad-hoc modifications, which we call “first aid strategies”, and bridged spatial distances by utilizing information systems.

As researchers and developers in this field, we were surprised how quickly even former critics of IT-based approaches began to boldly take initiative and transform their businesses in order to remain operative. One possible conclusion to this is that the global scale of the crisis beguiles decision makers to ignore necessary constraints and to reject a more comprehensive view in order to satisfy responsiveness. The pattern is evident in the heated discussion on tracing apps that suffered from a singular medical focus and developers are currently backtracking to make up for previously neglected concerns on personal privacy and data security. This suggests that ad-hoc implementations in particular can fail due to an oversimplified conception.

We see opportunities for BISE to rectify first aid strategies. Firstly, an important contribution can be the rigid and continuous pursuit of multi-perspectivity. This is reflected in the theories, models and methods that are being used regularly in the discipline and facilitate navigation to relevant and sound responses. Secondly, knowledge on the adaptation and projection of Information Systems artifacts (Baskerville and Pries-Heje 2019) could help to further increase both, responsiveness and thoroughness of first aid strategies, as it allows to draw on a plethora of well-developed examples.

### Crisis Intelligence

Once first measures have been taken, their substantiation necessitates a deeper understanding of causes and effects of the crisis. Certainly, one can argue that this step should be applied in the very beginning before taking any action. Yet, we see a hurdle for immediate crisis intelligence in the availability of data and limited accessibility for experts that investigate the effects of the crisis. Furthermore, strategies to enable the flexible selection and pooling of data within and across interest groups (e.g., along value chains) would also contribute to a more comprehensive view on the crisis. Here, BISE contributes to cooperation amongst experts within its very subject – the engineering and implementation of information systems. Expanding the possibilities of crisis intelligence thus requires the analysis and evaluation of new technologies and possibilities against the background of their practical value. Such can be the technical contribution to overarching, reliable cloud structures, e.g., the emerging federal *GAIA-X* initiative, as well as the managerial wayfinding for companies to publish datasets via public data ecosystems and platforms (Otto and Jarke 2019).

### Crisis Resilience

For us, a central goal of long-term solutions to crises lies in the structured recognition and mitigation of systemic risks (Mertens and Barbian 2015). This requires research that builds upon successful first aid strategies and crisis intelligence to increase the future resilience of value networks (Ivanov and Dolgui 2020). We find it noteworthy that many of the advances of BISE supersede genuine innovations as they can be reinterpreted in the light of the Corona crisis. For example, the virtual provision of services that has been examined as an innovative way to deliver maintenance and repair in manufacturing now enables the continuation of operations despite entry bans that are established in many countries. Here, resilience is drawn from insights about data-driven business models (Hartmann et al. 2016) and technology-enabled service strategies (Beverungen et al. 2019).

Even though this crisis enables BISE to contribute along the abovementioned avenues, this is not per se transferable to other crises. While Corona virus heavily affects analog living and working together, another crisis could disrupt the use of information systems (e.g., energy crises or data embargos). In that case, other disciplines may be in the position to remedy the immanent risks of information technology with their complementary skills. On that note, building up general crisis resilience would also mean identifying where information systems contain risks and to prepare first aid strategies in advance. Responsiveness and thoroughness are in this sense not antagonists: They rather intertwine when carefully implemented and suggest different courses of action for different situations.

In line with others before us, we see a leverage for crisis response in the involvement of different perspectives and the insights that can be gathered between and across scientific disciplines (Stember 1991; Shaw et al. 2018). Admittedly, this is not a new approach for BISE as a scientific field (Hasenkamp and Stahlknecht 2009), as it has the self-image of being interdisciplinary at its core (Mertens 1992). To us, however, the question of the demarcations of the discipline (e.g., the debate around Artificial Intelligence) becomes less important in the light of global crises. Instead, we advocate for the embracing of the variety of knowledge that can be utilized to study global crises and the creation of quick and substantiated collaborative responses.

## Business Informatics and Artificial Intelligence – The Silver Bullet to Fight COVID-19?

*Ingo Timm, Trier University and German Research Center for Artificial Intelligence (DFKI)*

### Introduction

Modern societies are increasingly interconnected through economic, social, and technological networks while modern means of transportation and mobility are marginalizing the distance between people, places, and countries. The travel time of information, goods, and people has been decreasing continuously over the last decades allowing for higher specialization with increasing efficiency of worldwide production and services networks. However, volatility, disturbances or errors are also quickly propagated in these networks, leading to *systemic risks* where small local events could potentially lead to situations getting out of control at a global level (Lorig et al. 2019). As technology, i.e., computer science and business informatics, has sped up networks and effects, the German Informatics Society (Gesellschaft für Informatik e.V.) has identified the control of systemic risks in worldwide networks as one of the grand challenges for the early 2020s. In the COVID-19 pandemic, it becomes obvious that even escalating infectious diseases have to be considered as systematic risks, which not only have an impact on public health, but affect almost all areas of human life. Thus, controlling pandemic scenarios is one of the great challenges for (business) informatics, especially for artificial intelligence.

### Pandemic Challenge: Managing the Crisis

The administrative reaction to a pandemic situation depends on political structures. The WHO specifies six phases covering three periods: inter-pandemic period, pandemic alert period, and pandemic period (WHO 2005). The global pandemic plan is supplemented by a national plan for Germany^2^ as well as further plans at a federal state level.^3^ On the regional level, districts with their health authorities are responsible for the local implementation of national or federal decisions and may have to decide on further local measures within their jurisdiction. Large-scale enterprises have to create emergency plans as well. However, these plans are mainly used for risk assessment and have a strong limitation: most of these plans are not really created for application and testing is not organized on a regular basis.

Yet, fighting a virus which becomes a global pandemic is a serious challenge and requires adaption of behavior and processes from almost every part of society. Not only from the government – being in charge of official regulations – but also the private and the public sector as well as the people themselves have to respond and act as quickly as possible. Immediate individual protection with a change of behavior has to be put in place even before all administrative regulations work. For example, at the very beginning of a pandemic, it is important that affected countries, regions, communities or companies respond to the first signs of the threat and take precautionary measures-even if the official pandemic declaration by the WHO is pending because it requires evidence of global spread.

**Fig. 1:**
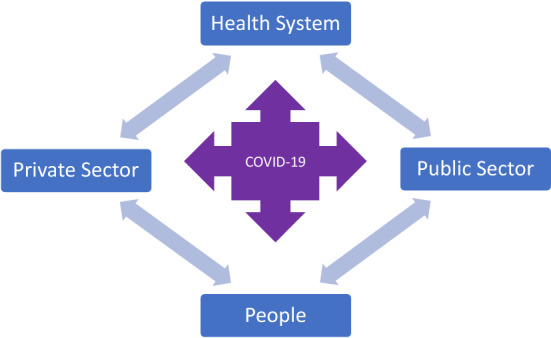
Crisis management dilemma between public health and social welfare

The organization and capacity of the health system play a crucial role. The situation becomes dramatic when it is no longer capable of nursing patients with severe symptoms. In a pandemic situation, public health, the capacity and burden of the health system on the one hand and the impact on the private and public sectors on the other hand have to be weighed against each other (Fig. 1).

This is a dilemma, as the areas are not independent of each other. Overburdening the health system will increase severe outcomes, deaths and prolongations of infection symptoms. As a consequence, numbers of available employees in the public and private sector will decrease and destabilize these sectors. Closing schools or kindergartens has similar effects on business and administration, however, this intervention has the potential to reduce the speed of transmission. Furthermore, business shut downs with interruption of systemic production and logistics chains weaken the health system through lack of drugs, instruments, hygienic material or food. Thus, a pragmatic approach is to focus on health care capacity and try not to eliminate transmission but to limit it in order to avoid overloading the health system. Decisions have to be made on enterprise, administrative, institutional levels, and by the people themselves. Since a pandemic situation is a rare event, there is a lack of experience and knowledge needed for decision-making. Therefore, new tools are needed to enable decision makers to make optimal decisions in the context of the dilemma described above, which is a challenge for research in AI and business information systems.

### Artificial Intelligence (AI) Fighting Corona

In the last decade, public interest in as well as expectations of AI have risen enormously. The manifold quick successes of machine learning (especially deep learning in various domains and applications) has led to an almost omnipresent demand of AI techniques. Therefore, it is not surprising that AI is on its way to fighting COVID-19. Data-driven AI in combination with Big Data is of specifically great importance. Naudé (2020) presents an initial review on different approaches of AI used against COVID-19 in the following six areas: (i) early warnings and alerts, (ii) tracking and prediction, (iii) data dashboards, (iv) diagnosis and prognosis, (v) treatment and cures, and (vi) social control. While Google Flu Trends have not been specific enough for practical application (Lazer er al. 2014), *Bluedot*^4^ or *HealthMap*^5^ succeeded in predicting the outbreak of the infection in late 2019. Data-driven AI is part of the regular research in eHealth in the areas of data dashboards, diagnosis and prognosis, treatment and cures. The applications range from the analysis of CT images or blood samples to interactive chat bots. As pandemics are "rare events", availability of data and experience is one of the main limitations here. COVID-19-relevant AI applications can be divided into two types: On the one hand, there are many approaches reinforcing other COVID-19-relevant sciences based on long-term successful interdisciplinary cooperation, such as deep learning in imaging techniques. On the other hand, AI researchers use freely available data or knowledge to demonstrate the impact of their approaches to COVID-19 aspects as an example of a real-world application. Many AI approaches are tested in this way, but for practical application, mediation processes between AI experts and decision makers with their specific expertise and information needs are necessary.

### Business Information Systems Fighting Corona

From a business informatics perspective, information management as well as decision support are under question for supporting crisis management. Pandemic crisis situations – being one specific type of systemic risk – require competent scientific advice on possible measures and their effects on infection transmission as well as advice on economic and social consequences. Availability, quantity, and quality of information change during pandemics. In the beginning, there is little reliable data and knowledge available. Expertise from different domains have to be included in decision making, e.g., virology, pharmacy, epidemiology, biometrics, statistics, social sciences, or psychology. Furthermore, information and knowledge can be acquired or generated, e.g., by infection scouts, smartphone apps or sharing of offline data. However, resources are limited and costs as well as utility have to be balanced. Business information systems have to be engineered to ensure availability, situational aggregation, and interpretation of decision-relevant information. However, decision support via conventional prediction models, e.g., by analyzing and evaluating alternative courses of action, are of limited significance as data and experience are missing. In many areas of information systems research, especially production and logistics, computer simulation is an important component for complex systems (Hudert 2010). However, transmission in pandemics is highly dependable on people’s individual intent and decisions, their interaction with other people, as well as their social environment. For example, the unexpected high demand for toilet paper can hardly be predicted by means of statistics while it can be explained with social theories like social congestion. Agent-based social simulation for analyzing such complex social systems, agent-based social simulation (ABSS) has been successfully proven to, e.g., (Berndt et al. 2018). ABSS has great potential in this context. When applying theories and methods distributed by AI, these simulations can be extended not only by social but also cognitive models, so that a comprehensible behavior is created. Furthermore, the models can utilize expert knowledge, like rules and mechanisms. ABSS becomes feasible and useful at the beginning of a pandemic when data availability or quality is weak. Consequently, there are interesting simulation approaches to Corona pandemics in development, e.g., Agent-based Social Simulation of the Corona virus Crisis (ASSOCC)^6^ or Social Simulation for Infectious Disease Control (SoSAD).^7^

### Pandemics: The Hour of Combined Approaches of AI and Business Informatics

In summary, the COVID-19 pandemic is a major challenge for society and a particular challenge for AI and business informatics. AI by itself can deliver important contributions to the fight against COVID-19. However, it is not a silver bullet: applications and approaches must be carefully chosen. Whether in the the decision-making phase of a lock down or the current phase of relaxing or reintroducing of measures, there is a great need for information and advice in districts and for their health authorities. This is a typical task for business informatics. The documentation and tracing of infection paths, the data and information exchange with other offices and regions, as well as the weighing of measures and their effects should be supported by knowledge-based business information systems and computer simulations. Thus, an integrated approach of AI and business informatics plays a key role in the regional fight against COVID-19.

## Footnotes

1 https://mipasa.org/about/.

2 Robert Koch Institut (RKI). *Nationaler Pandemieplan Teil I – Strukturen und Maßnahmen.* Robert-Koch-Institut, March 2nd, 2017. https://www.gmkonline.de/documents/pandemieplan_teil-i_1510042222_1585228735.pdf.

3 https://www.rki.de/DE/Content/InfAZ/I/Influenza/Pandemieplanung/Pandemieplaene_Bundeslaender.html.

4 https://bluedot.global.

5 https://healthmap.org.

6 Dignum, F. et al*.*: https://simassocc.org/.

7 Timm, I.J. et al*.*: https://sosad.wi1.uni-trier.de/.
